# Maintaining Weight Loss in Obese Men with Prostate Cancer Following a Supervised Exercise and Nutrition Program—A Pilot Study

**DOI:** 10.3390/cancers13143411

**Published:** 2021-07-07

**Authors:** Rebekah L. Wilson, Dennis R. Taaffe, Robert U. Newton, Nicolas H. Hart, Philippa Lyons-Wall, Daniel A. Galvão

**Affiliations:** 1 Division of Population Sciences, Department of Medical Oncology, Dana-Farber Cancer Institute, Boston, MA 02215, USA; rebekahl_wilson@dfci.harvard.edu; 2 Exercise Medicine Research Institute, Edith Cowan University, Perth, WA 6027, Australia; d.taaffe@ecu.edu.au (D.R.T.); r.newton@ecu.edu.au (R.U.N.); nicolas.hart@qut.edu.au (N.H.H.); p.lyons-wall@ecu.edu.au (P.L.-W.); 3 School of Medical and Health Sciences, Edith Cowan University, Perth, WA 6027, Australia; 4 Cancer and Palliative Care Outcomes Centre, Queensland University of Technology, Brisbane, QLD 4000, Australia

**Keywords:** nutrition, exercise, body composition, physical function, androgen deprivation therapy, home-based

## Abstract

**Simple Summary:**

More than 50% of prostate cancer patients will receive androgen deprivation therapy (ADT) and 70% will experience ADT-induced weight gain. Supervised exercise and nutrition interventions are viable strategies to mitigate or reverse ADT-induced body composition changes; however, the ability to preserve these benefits when supervision is no longer available is unclear. Our study examined the effects of a home-based weight maintenance program on body composition and physical function in obese men with prostate cancer on ADT who had previously completed a supervised weight loss intervention. We demonstrated that a home-based weight maintenance program can preserve body composition and physical function for at least 12 weeks following a supervised intervention. This study provides insight into the prospect of home-based programs to preserve benefits gained within a supervised environment for patients remaining on ADT when ongoing in-person services are no longer viable.

**Abstract:**

Supervised exercise and nutrition programs can mitigate or reverse androgen deprivation therapy (ADT) induced fat mass (FM) gain, lean mass (LM) loss, and impaired physical function. It is unclear whether these benefits are retained following transition to self-management. This study examined the effect of a home-based weight maintenance program on body composition and physical function in obese men with prostate cancer (PCa) on ADT following a 12-week supervised weight loss intervention. Eleven obese PCa patients (74 ± 5 years, 40.0 ± 4.9% body fat) on ADT (>6 months) completed a 12-week self-managed home-based weight maintenance program consisting of 150 min/week of aerobic and resistance training while maintaining a healthy balanced diet. Body composition (DXA), muscle strength (1RM), and cardiorespiratory fitness (400 m walk) were assessed. Significant reductions in weight (−2.8 ± 3.2 kg) and FM (−2.8 ± 2.6 kg), preservation of LM (−0.05 ± 1.6 kg), and improvements in muscle strength and VO_2max_ were achieved across the supervised intervention. Across the home-based program, no significant changes were observed in weight (−0.6 ± 2.8 kg, *p* = 0.508), FM (0.2 ± 1.4 kg, *p* = 0.619), LM (−0.8 ± 1.6 kg, *p* = 0.146), muscle strength (−0.2 to 4.1%, *p* = 0.086–0.745), or estimated VO_2max_ (0.3 ± 2.1 mL/min/kg, *p* = 0.649). Self-managed, home-based exercise and nutrition programs are a viable strategy to promote maintenance of body composition and physical function following a supervised intervention in obese PCa patients on ADT.

## 1. Introduction

Improvements in screening procedures and medical treatments have elevated prostate cancer to a chronic condition where ~90% of patients with localised disease survive more than 10 years beyond diagnosis [1,2]. While beneficial, increasing survival rates are accompanied by long-term treatment and cancer-related adverse effects that can negatively impact quality of life [3,4]. Androgen deprivation therapy (ADT) is a common neoadjuvant, adjuvant, and primary treatment for prostate cancer, prescribed for months or years, intermittently or indefinitely [5]. Adverse changes in body composition are a common side effect of ADT with previous reports indicating a 6.6 to 13.8% gain in fat mass (FM), 2.0 to 3.6% loss in lean mass (LM), and 2.0 to 8.0% loss in bone mass within the first year of treatment [6,7]. Fat mass gain is associated with faster development of castrate resistance, an increased risk of fatal prostate cancer, and development of obesity-related comorbidities [8,9,10]. Loss of LM and bone mass can reduce physical function and increase risk of falls and fractures, which may lead to increased morbidity [11]. More than half of men with prostate cancer are likely to receive ADT at some point during their cancer journey [5], so it is important to determine appropriate adjuvant therapies to prevent or regulate long-term ADT-induced side effects.

Exercise and nutrition programs are often provided as adjuvant therapies for men with prostate cancer [12]. While supervised interventions are typically more effective due to face-to-face instruction and personal accountability resulting in high adherence [13], home-based unsupervised or minimally supervised programs are of interest to some clinicians and patients to reduce costs and allow increased access to people who cannot travel frequently to an exercise venue [14,15]. Two exercise and nutrition-based interventions by Bourke et al. [16,17] examined a self-managed period after progressively reducing supervision for men with prostate cancer on ADT compared to usual care controls. Both studies demonstrated an improvement in aerobic fitness and exercise and nutrition behaviour during the supervised intervention, which were maintained during the non-supervised follow-up period, with Bourke, et al. [16] also reporting improvements in muscle strength. While these studies provided preliminary feasibility for a self-managed period to preserve aerobic fitness, muscle strength, and exercise and nutrition behaviours, neither study targeted obese men with prostate cancer or reported an intervention effect on body composition-related measures during the supervised or non-supervised period. This needs to be further investigated, particularly as their programs only examined anthropometric measures.

Previously we have reported preliminary efficacy of a 12-week supervised exercise and nutrition weight loss intervention to significantly reduce FM and maintain LM in obese men with prostate cancer receiving ADT, with improvements in muscle strength and cardiorespiratory fitness also observed [18]. Weight regain is common after a weight loss intervention [19]. Additionally, this patient population may be at increased risk of weight regain due to ongoing ADT [6,7] and it is unclear if a self-managed home-based exercise and nutrition program is a viable approach to maintain previously established benefits in body composition and physical function after a period of effective supervision. Therefore, this pilot study examines the effect of a 12-week self-managed home-based exercise and nutrition program on body composition and physical function in obese men with prostate cancer on ADT, following completion of the supervised weight loss intervention. We hypothesized that a 12-week home-based exercise and nutrition program would preserve the body composition and physical function improvements achieved subsequent to the supervised exercise and nutrition program. 

## 2. Materials and Methods

### 2.1. Study Design and Participants

This is a follow-up report to a self-controlled prospective study of a supervised exercise and nutrition weight loss intervention [18]. Eleven obese men with prostate cancer completed the 12-week supervised intervention and continued with the presently examined self-managed home-based program. Details of inclusion criteria, recruitment, and study design for the self-controlled prospective study have been previously reported [18]. Briefly, patients completed a 6-week control period undertaking their normal activities followed by a 12-week supervised weight loss intervention that included combined aerobic and resistance training 3 times per week, and individualised nutrition advice to establish an energy deficit of 2100–4200 kJ per day (d). Men were also provided with a 40 g whey protein supplement after each supervised exercise session (Whey protein concentrate, Bulk Nutrients, TAS, Australia). The study was approved by the Edith Cowan University Human Research Ethics Committee (ID: 18832). All patients provided written informed consent. 

### 2.2. Home-Based Program

Patients were advised to complete 150 min of combined aerobic and resistance training each week, while maintaining a healthy balanced diet based on the Australian Dietary Guidelines [20]. The lead researcher (RLW) facilitated the transition from a supervised weight loss intervention to a self-managed home-based weight maintenance program, by providing an information booklet and a single face-to-face training session of resistance exercises to be completed at home with use of a GYMSTICK^TM^ (Ratavartijankatu, Finland). Examples of aerobic and resistance exercises to be completed at moderate-to-vigorous intensity, and strategies to maintain a healthy balanced diet were included in the booklet. Patients attended a nutrition counselling session immediately following the supervised intervention where individual goals established at the start of the supervised weight loss intervention were reassessed and adapted based on the patient’s weight loss progress, with weight maintenance being the general goal. As a protein supplement was not provided for the home-based program, advice was given to maintain an adequate protein intake (1.07 g/kg body weight/d [21]). Patients were contacted once by telephone at week 6 of the 12-week home-based program to address any questions or concerns about maintaining their exercise and nutrition regimen. All tests were conducted at post-supervised intervention (week 1 of home-based program) and after week 12 of the home-based program, unless otherwise stated.

### 2.3. Measurements

#### 2.3.1. Body Composition 

Total body mass (kg), FM (kg), bone-mineral free LM (kg), body fat percent (%), trunk FM (kg), visceral FM (g), appendicular skeletal muscle (ASM, kg), and bone mineral content (BMC, g) were assessed by dual-energy x-ray absorptiometry (DXA). ASM was calculated as the sum of upper limb and lower limb LM [22]. Waist and hip circumference (cm) were measured according to standardised procedures with a constant-tension tape measure [23].

#### 2.3.2. Muscle Strength and Cardiorespiratory Fitness

Muscle strength was assessed by one-repetition maximum (1RM) for the chest press, leg press (seated or incline), and seated row exercises [24], and cardiorespiratory fitness (VO_2max_) was estimated by the 400 m walk test [25]. Estimated VO_2max_ was calculated using the following equation and is highly correlated (r = 0.83) with directly measured peak VO_2_ in men [25]:

VO_2_ = 39.431 − (0.054 × 400 m time) + (2.832 × long stride) − (0.031 × end SBP) − (0.064 × CF)

where 400 m time is in seconds, long stride is 1 for stride <1.2 steps/m or 0 for stride >1.2 steps/m, end SBP is measured in mmHg, and CF refers to the correction factor, which, if the time taken to perform the 400 m course is slower than 240 s, is 0, but if the time is faster than 240 s, then the CF is time in seconds to complete the 400 m minus 240.

#### 2.3.3. Resting Metabolic Rate

Resting metabolic rate (RMR, kcal/d) was measured in the morning via indirect calorimetry using a canopy hood (Fitmate, COSMED, Rome, Italy) [26]. Prior to arrival, patients were instructed to complete a minimum 10-h overnight fast with allowance for water and morning medications. On arrival, patients rested in a supine position in a darkened room for 10 min, after which the ventilated hood was placed over their head and secured to avoid leakage of gases. Exhaled breath was collected until sufficient data were collected for analysis or until 10 min, whichever occurred first.

#### 2.3.4. Physical Activity Monitoring

Physical activity and sedentary behaviour were objectively assessed using the ActiGraph wGT3X-BT accelerometer (ActiGraph LLC, Pensacola, FL, USA). Patients were instructed to wear the accelerometer on their hip continuously for 24 h/d for 3 consecutive days (1 weekend day and 2 weekdays), excluding water-based activities, with ActiLife software used for analysis (ActiLife 6, ActiGraph LLC, Pensacola, FL, USA). Only wake wear time was used with a minimum data collection period required for inclusion in the analysis set at 1 day of at least 600 min. Non-wear time was excluded from analysis and defined as 90 min or more of consecutive zeros with a 2-min spike tolerance [27]. Commonly used cut-off points among cancer patients were used to classify sedentary time (<100 counts per min, cpm), light physical activity (100–1951 cpm), and moderate-to-vigorous physical activity (≥1952 cpm) [28,29,30]. The modified Godin Leisure-Time Exercise Questionnaire was also completed pre and post the home-based program to assess the average time spent undertaking resistance training during a typical week in the previous month [31].

#### 2.3.5. Nutrition Monitoring

Patients completed a 3-day weighed food record (3d-WR) comprising 1 weekend day and 2 weekdays. This information was used to estimate the average daily intake of energy (kJ) and macronutrients. The 3d-WR was analysed using FoodWorks dietary analysis software (FoodWorks 10 Professional, Xyris Software Pty Ltd, Brisbane, QLD, Australia).

### 2.4. Statistical Analysis

Sample size was calculated based on the anticipated fat mass changes to occur during the supervised intervention and has been previously described [18]. Briefly, to achieve 90% power at an α level of 0.05 (two-tailed) in a single-group study and account for an attrition rate of up to 15%, 14 participants were required to detect a ≥2 kg reduction in fat mass. Data were analysed using IBM SPSS version 25 (SPSS Inc., IBM Corp, Armonk, NY, USA). The Shapiro–Wilk test was used to determine normality of the distribution. Paired *t*-tests were used to compare normally distributed variables between post-supervised intervention and post-home-based program, while the Wilcoxon signed rank test was used for non-normally distributed data. Pearson’s correlation or Spearman’s rank correlation were used to assess associations, as appropriate. Data are presented as mean ± standard deviation (SD), median and interquartile range [IQR], or number (percentage). All tests were two-tailed with statistical significance set at *p* < 0.05.

## 3. Results

Eleven patients aged 63 to 82 years completed the 12-week self-managed home-based program (Table 1). More than half of the men had a Gleason score of 9 (54.5%), with 54.5% of patients also diagnosed with metastatic cancer in the lymph nodes or visceral organs at study entry. During the home-based program, one patient developed nodal metastases resulting in an anti-androgen being prescribed, and a second patient developed metastases to their adrenal glands resulting in further radiation therapy.

### 3.1. Nutrition and Physical Activity

A significant increase in total energy intake was observed from post-supervised to post-home-based program with a median change from 6759 to 7972 kJ/d (*p* = 0.041) (Table 2). Carbohydrate intake (179.8 ± 68.0 vs 206.1 ± 67.1 g/d, *p* = 0.016) was also significantly increased across the home-based period. There were no significant changes in the percentage of energy derived from protein, total fat, carbohydrate, or alcohol, and intakes were within Acceptable Macronutrient Distribution Ranges [21]. There was a significant increase from post-supervised to post-home-based program in the percentage of wake time spent in sedentary behaviour (65.9 to 70.2%, *p* = 0.003) and a significant decrease in the average time and percentage of wake time spent in light physical activity (4.8 to 4.0 h/day, *p* = 0.011; 33.3 to 28.6%, *p* < 0.001, respectively) (Table 2). Additionally, self-reported resistance training duration decreased from 143 to 113 min/week but was not significantly different (*p* = 0.685). 

### 3.2. Body Composition 

**Figure 1:**
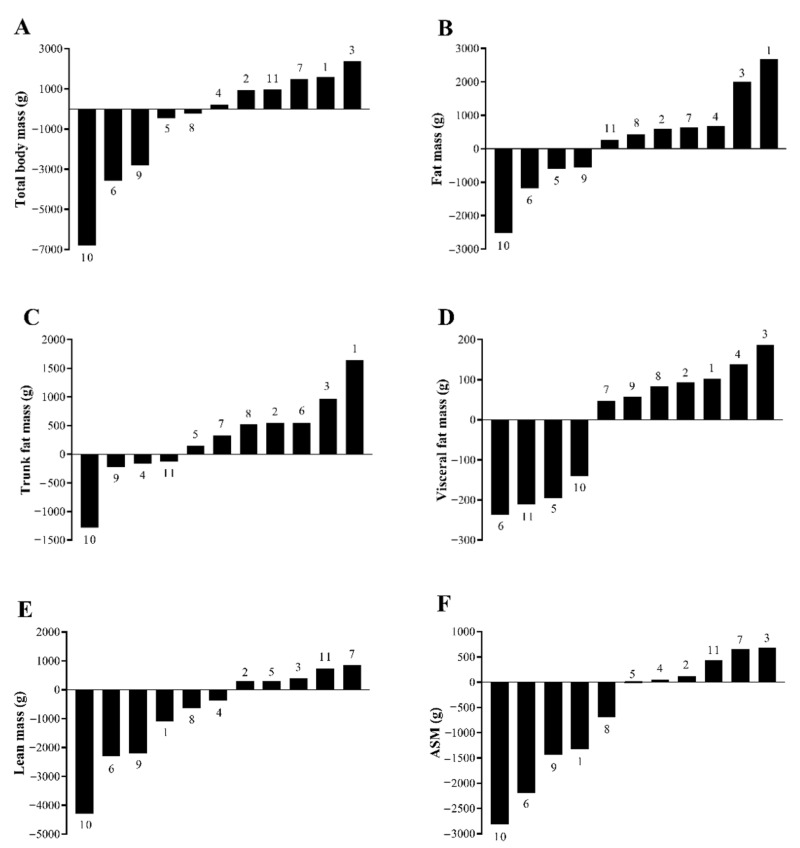
Waterfall plots of individual patient changes over a 12-week self-managed home-based program presented in ascending order for: (**A**) total body mass, (**B**) total fat mass, (**C**) trunk fat mass, (**D**) visceral fat mass, (**E**) total lean mass, and (**F**) appendicular skeletal muscle (ASM) mass. Individual patient numbers are identified in association with the bars.

No significant changes were observed in body mass or any body composition measure except for a modest increase in body fat percent (0.6 ± 0.8%, *p* = 0.034) from post-supervised to post-home-based program (Table 3). Individual changes in body composition are presented in Figure 1. During home-based follow-up, four (36.4%) patients lost FM and five (45.5%) patients gained LM. Correlation analysis indicated resistance training duration (min/week) was significantly associated with change in LM (r_s_ = 0.606, *p* = 0.048). No significant associations were found for absolute values or change in energy intake (r_s_ = −0.091–0.173, *p* = 0.612–0.811), sedentary time, light physical activity, and protein intake per kg of body weight with change in FM or LM (r = −0.316–0.438, *p* = 0.178–0.928).

### 3.3. Muscle Strength, Cardiorespiratory Fitness, and Resting Metabolic Rate

No significant changes in chest press strength (*p* = 0.745) or seated row strength (*p* = 0.744) were observed; however, leg press strength approached significance (*p* = 0.086) with a 6.1 kg increase reported across the home-based program (Table 4). No significant changes were observed for RMR (*p* = 0.450) or cardiorespiratory fitness as determined by estimated VO_2max_ (*p* = 0.640) (Table 4).

### 3.4. Adverse Events

One patient experienced a muscle strain in their chest and gluteal muscles in the final week of the home-based program while completing the prescribed exercise. This precluded him from completing the chest and leg press strength 1RM at the post-test session. No other study-related adverse events were reported while undertaking study-related activities. However, four patients experienced adverse events associated with pre-existing comorbidities and musculoskeletal conditions. One patient was hospitalised with a septic wound on their foot and could not complete any physical function testing following the home-based program. Three patients experienced progressive pain in the knee, back, shoulder, or ankle that prevented the completion of the leg press 1RM at the post-home-based program testing session. Each patient received medical care and reduced their activity levels as per clinician recommendations. 

## 4. Discussion

In this research, we examined the effect of a self-managed home-based weight maintenance program in obese men with prostate cancer on ADT following a self-controlled prospective study of a supervised weight loss intervention. There were two main findings: (1) total and regional FM and LM were preserved; and (2) muscle strength and cardiorespiratory fitness were preserved. 

Weight regain in obese men is common after intentional weight loss [19] and is likely to be exacerbated by ADT. As ADT can be prescribed for years or indefinitely, it is important to examine exercise and nutrition intervention strategies that may help mitigate treatment-related side effects. This study showed that a self-managed home-based exercise and nutrition program can promote weight maintenance by preserving FM for at least 12 weeks after a supervised intervention in obese men with prostate cancer while on ADT who had previously benefited from a supervised weight loss intervention. This occurred irrespective of a reduction in physical activity and an increase in energy intake suggesting that the FM previously lost across a supervised intervention may be maintained with a lower workload. Freedland, et al. [32] also examined overweight and obese men with prostate cancer on ADT and compared a home-based intervention to usual care. While it was a home-based program, their intention was to induce weight loss, not preserve body weight as in our current study, thus changes across intervention groups are not directly comparable. However, the usual care control group in the Freedland, et al. [32] study demonstrated a 10.9% increase in FM, which was statistically different to the intervention group, compared to a non-significant 1.1% FM increase in the present study. Our study duration was 12 weeks and engaged men who had been on ADT for a minimum of 6 months, whereas the Freedland, et al. [32] study was 6 months in duration and engaged men initiating ADT. Patients initiating ADT experience substantial changes in body composition in the first few months that slowly plateaus within the first year and FM remains elevated if no intervention is implemented [33,34]. This may account for the larger changes in the usual care control group of the Freedland, et al. [32] study. Nonetheless, the comparatively small changes observed in our study demonstrate potential for home-based exercise and nutrition programs to preserve FM in obese patients on ADT for at least 12 weeks following a period of supervised weight loss. However, the results of the present study may not be applicable to a population who has not undertaken a supervised exercise intervention as the participants examined here were likely more motivated to continue with the recommended lifestyle behaviours previously introduced in a supervised environment that resulted in observable benefits [18].

Lean mass is the predominant contributor to RMR [35,36]; therefore, the maintenance of LM is vital for long-term weight management. While there was no significant change in LM, suggesting overall continued preservation from the supervised intervention, there was a mean 0.8 kg decrease over the 12-week home-based program. Had the follow-up period been longer, LM could have continued to decline resulting in a different statistical outcome. This highlights the value of the inclusion of strength training as a potentially critical component for LM preservation in this patient population when completing home-based exercise. This was a small sample size, so correlation analysis has limited applicability; however, our preliminary analysis showed the duration of self-reported resistance exercise was positively correlated with LM change. No relationship between LM change and the changes in energy intake, sedentary behaviour, light physical activity, and protein intake were found. All patients were provided with a GYMSTICK^TM^ as well as actively encouraged to join a gym or fitness group to undertake resistance training. How patients distributed their resistance training practices, e.g., utilising resistance machines, free weights, and GYMSTICK^TM^, is unknown due to a lack of returned activity logs. While it has been suggested that there are no superior strength gains when using conventional resistance equipment compared to elastic-based resistance equipment [37], their differing effects on LM is unclear. This may further explain the trend for a decline in LM as the use of a GYMSTICK^TM^ alone, in addition to the removal of the protein supplement provided during the supervised period, may not have satisfied the necessary threshold to stimulate muscle protein synthesis when transitioning from a clinic-based supervised weight loss intervention to a home-based weight maintenance program. 

Several patients in our study experienced an injury or illness related to known pre-existing comorbidities and musculoskeletal conditions during the home-based program; consequently, we further explored the individual changes that occurred across the two intervention phases to better understand how these events may be addressed in future research. For example, patient #6 achieved a desirable FM loss (₋5.3 kg) and LM gain (2.6 kg) during the supervised intervention [18]. However, due to progressive deterioration of a musculoskeletal condition, this patient reported avoidance of exercise during the home-based program. Although patient #6 lost further FM (₋1.2 kg), they reversed their LM gain with a 2.3 kg loss over the 12-week home-based program. Similar patterns were noted for patients #4 and #9. In contrast, patient #3 had a desirable FM loss (₋7.1 kg), but also lost LM (₋2.3 kg) during the supervised intervention. During the last 4 weeks (12 sessions) of the supervised intervention, this patient missed six sessions due to illness and had to complete four sessions at a reduced intensity. However, with the implementation of exercise at home, this patient prevented further LM loss with a 0.4 kg gain over the 12-week period. These individual changes raise questions about the feasibility of LM maintenance in injured or ill patients who may not be able to undertake sufficient exercise to stimulate muscle protein synthesis, particularly resistance training. Such patients in a supervised environment have assistance readily available to them to adjust their exercise prescription as required, whereas those undertaking self-managed exercise do not. Other LM management strategies could include protein supplementation and regular video or telephone consultations via telehealth to monitor adherence and compliance and assist with exercise program modification to account for injury or illness if in-person supervision is not viable or desired. 

Prolonged ADT significantly impacts the musculoskeletal system placing men with prostate cancer at increased risk of disability [38]. Additionally, patients typically reduce their physical activity levels because of severe treatment-related side effects such as fatigue, reduced physical function, or urinary incontinence [39], which can lead to diminished cardiorespiratory fitness. For this reason, it is important to ensure muscular strength and cardiorespiratory fitness are maintained. In the current follow-up study, patients in a self-managed home-based program maintained upper- and lower-body muscle strength, walking endurance, and cardiorespiratory fitness irrespective of a decline in LM. Previous studies using home-based programs following supervised exercise and nutrition [16,17] or exercise-only [40] programs also reported maintenance of muscle strength and cardiorespiratory fitness, which is reflected in the results of our study. The present study extends these findings by demonstrating that obese patients on long-term ADT can also preserve their muscle strength and cardiorespiratory fitness using a home-based program following a supervised intervention. 

This study has several strengths. First, DXA allowed the evaluation of whole-body and regional changes in FM and LM. Second, this study reports on a novel cohort, that is, obese men with prostate cancer on long-term ADT and their response to a self-managed home-based exercise and nutrition program following supervised exercise and dietary advice. Limitations include the use of a small single group cohort with no control group comparison, although a control period was undertaken prior to the supervised component and the study was powered for the primary outcome of FM [18]. Four patients were not able to complete all measures of physical function at the post-home-based program due to poor physical health. Implementing a self-managed home-based exercise and nutrition program in future studies could benefit from increased frequency of contact with patients to ensure exercise and nutrition modifications reflect any changes to their health status. Daily physical activity logbooks were poorly kept by patients and as such were not utilised. However, the comparison of self-reported resistance exercise from the modified Godin Leisure-Time Exercise Questionnaire, physical activity data collected using ActiGraph, and nutritional intake assessed using the 3d-WR at post-supervised and post-home-based program, provided important insight into the behaviour changes undertaken.

## 5. Conclusions

This pilot study provides preliminary evidence that obese men with prostate cancer receiving ADT, who previously benefited from a supervised weight loss exercise and nutrition intervention, can maintain their body composition and physical function improvements by undertaking a self-managed home-based exercise and nutrition program. However, maintenance of LM was dependent on the duration of weekly resistance exercise and should be an important consideration for future programs. As ADT may be prescribed for several months, years or even indefinitely, it is important to implement interventions such as exercise and nutrition programs that may prevent or regulate ADT side effects and improve patient outcomes. Home-based programs will likely play an important role in maintaining the positive effects gained from clinic-based programs once supervision is no longer available or feasible for the patient. However, further research is required to investigate the feasibility of self-managed programs for obese people with cancer who are likely to have multiple comorbidities placing them at increased risk of illness and injury. From this study, we provide the foundation for larger scale interventions to further examine long-term adherence and compliance, and whether obese people with cancer can continue to effectively manage ongoing treatment-related adverse effects, in particular FM gain and LM loss.

## Figures and Tables

**Table 1:** Patient characteristics at post-supervised intervention.

Variable	Patients (*n* = 11)
Age (years), mean ± SD	74 ± 5
Body mass index (kg/m^2^), mean ± SD	33.1 ± 5.3
Post-secondary education, *n* (%)	8 (72.7)
Married, *n* (%)	11 (100)
Employed, *n* (%)	1 (9.1)
Current smoker, *n*	0
Number of medications/supplements, mean ± SD	4.5 ± 2.9
Number of comorbidities, mean ± SD ^a^	3.4 ± 1.4
Years since prostate cancer diagnosis, median [IQR]	3.9 [1.5–9.7]
Gleason score, *n* (%)	
Gleason 7	3 (27.3)
Gleason 8	1 (9.1)
Gleason 9	6 (54.5)
Gleason 10	1 (9.1)
Contained within prostate, *n* (%)	5 (45.5)
Lymph node metastasis, *n* (%)	4 (36.4)
Organ metastasis, *n* (%) ^b^	2 (18.2)
Androgen deprivation therapy, *n* (%)	
LHRH agonist + antiandrogen	7 (63.6)
LHRH agonist only	4 (36.4)
Months on ADT, median [IQR]	16 [9,10,11,12,13,14,15,16,17,18,19,20,21,22,23,24,25,26,27]
Other prostate cancer-related treatment, *n* (%)	
Surgery	4 (36.4)
Radiation therapy	10 (90.9)
Chemotherapy	2 (18.2)

^a^ Types of comorbidities: Arthritis, atrial fibrillation, cardiovascular disease, carpel tunnel syndrome, colitis, dyslipidaemia, hypertension, sleep apnoea, thyroid disease, emphysema, type 2 diabetes, peripheral neuropathy. ^b^ Lung, adrenal gland. LHRH—luteinizing hormone-releasing hormone; ADT—androgen deprivation therapy.

**Table 2:** Nutrition intake as assessed by 3d-WR and physical activity as assessed by ActiGraph at post-supervised intervention and post-home-based program.

Variable	Post-Supervised Intervention	Post-Home-Based Program	Mean Change	*p*-Value
Nutrition intake				
Energy intake (kJ/d)	6759 [4994–8980]	7972 [6353–8535]	-	0.041
Protein (g/d)	85.9 ± 24.3	93.8 ± 21.1	8.0 ± 20.3	0.222
Protein (% total energy)	21.2 ± 4.0	20.1 ± 2.9	−1.1 ± 4.5	0.433
Fat (g/d)	60.7 ± 21.8	67.7 ± 20.9	7.0 ± 24.1	0.360
Fat (% total energy)	31.5 ± 4.8	31.0 ± 4.8	−0.5 ± 6.7	0.823
Carbohydrate (g/d)	179.8 ± 68.0	206.1 ± 67.1	26.2 ± 29.9	0.016
Carbohydrate (% total energy)	40.3 ± 4.3	40.7 ± 6.1	0.4 ± 5.9	0.848
Alcohol (% total energy)	0.0 [0.0–3.0]	2.1 [0.0–6.8]	-	0.310
Physical activity				
Average time in SB (h/d)	9.6 ± 1.9	9.5 ± 2.0	−0.1 ± 2.0	0.871
Time spent in SB (% wake hours)	65.9 ± 6.2	70.2 ± 8.8	4.3 ± 3.8	0.003
Average time in LPA (h/d)	4.8 ± 1.2	4.0 ± 1.6	−0.9 ± 0.9	0.011
Time spent in LPA (% wake hours)	33.3 ± 6.3	28.6 ± 8.1	−4.8 ± 3.1	<0.001
Average time in MVPA (min/d)	6.9 ± 5.4	10.7 ± 10.1	3.8 ± 11.2	0.286
Time spent in MVPA (% wake hours)	0.6 [0.3–1.1]	0.7 [0.3–2.2]	-	0.424

Values are the mean ± SD where a paired *t*-test was used or median [IQR] where a Wilcoxon signed rank test was used. SB—sedentary behaviour; LPA—light physical activity; MVPA—moderate to vigorous physical activity.

**Table 3:** Body composition and anthropometric changes over the 12-week home-based program.

Variable	Post-Supervised Intervention	Post-Home-Based Program	Mean Change	Percent Change (%)	*p*-Value
Total body mass (kg)	95.5 ± 14.1	94.9 ± 12.9	−0.6 ± 2.8	−0.4 ± 2.7	0.508
Total fat mass (kg)	37.0 ± 9.5	37.3 ± 8.7	0.2 ± 1.4	1.1 ± 4.0	0.619
Percent body fat (%)	38.3 ± 4.6	38.9 ± 4.5	0.6 ± 0.8	-	0.034
Trunk fat (kg)	18.3 ± 5.4	18.5 ± 5.2	0.3 ± 0.7	2.0 ± 4.5	0.271
Visceral fat (g)	866 ± 333	860 ± 277	−7 ± 156	−1.6 ± 17.0	0.888
Total lean mass (kg)	55.9 ± 6.2	55.1 ± 6.2	−0.8 ± 1.6	−1.3 ± 2.7	0.146
ASM (kg)	23.3 ± 3.1	22.7 ± 3.1	−0.6 ± 1.2	−2.5 ± 4.9	0.130
BMC (g)	2576 ± 291	2544 ± 261	−32 ± 56	−1.1 ± 2.0	0.087
Waist circumference (cm)	103.9 ± 8.9	103.5 ± 8.5	−0.4 ± 2.6	−0.3 ± 2.5	0.626
Hip circumference (cm)	109.7 ± 8.1	108.7 ± 7.9	−1.0 ± 2.2	−0.9 ± 1.9	0.141

Values are the mean ± SD. ASM—appendicular skeletal muscle; BMC—bone mineral content.

**Table 4:** Changes in muscle strength, cardiorespiratory fitness, and resting metabolic rate over the 12-week self-managed home-based program.

Variable	Post-Supervised Intervention	Post-Home-Based Program	Mean Change	*p*-Value
Leg press (kg) (*n* = 7)	126.3 ± 47.4	132.4 ± 53.7	6.1 ± 7.9	0.086
Chest press (kg) (*n* = 8)	51.8 ± 14.1	51.0 ± 13.0	−0.7 ± 6.0	0.745
Seated row (kg) (*n* = 10)	68.2 ± 7.9	68.8 ± 8.9	0.6 ± 5.4	0.744
Estimated VO_2max_ (mL/min/kg) (*n* = 9)	20.6 ± 3.6	20.3 ± 3.3	−0.3 ± 1.6	0.602
RMR (kcal/d) (*n* = 11)	1516 ± 207	1482 ± 186	−34 ± 143	0.450

Values are the mean ± SD. VO_2max_—maximal oxygen consumption; RMR—resting metabolic rate.

## Data Availability

The data that support the findings of this study are available from the corresponding author upon reasonable request.
